# Preparation of Sustainable Alginate/Chitosan Blend Films by Thermo-Compression for Diverse Applications

**DOI:** 10.3390/gels12010063

**Published:** 2026-01-09

**Authors:** Yodthong Baimark, Prasong Srihanam, Theeraphol Phromsopha, Nuanchai Khotsaeng

**Affiliations:** 1 Biodegradable Polymers Research Unit, Department of Chemistry and Centre of Excellence for Innovation in Chemistry, Faculty of Science, Mahasarakham University, Maha Sarakham 44150, Thailand; prasong.s@msu.ac.th (P.S.); theeraphol.p@msu.ac.th (T.P.); 2 Faculty of Science and Health Technology, Kalasin University, Namon District, Kalasin 46230, Thailand; nuanchai.k@ksu.ac.th

**Keywords:** alginate, chitosan, blend films, thermo-compression, mechanical properties, water resistance

## Abstract

In this work, sodium alginate/chitosan (SA/CS) blend films were prepared by thermo-compression for the first time. Glycerol and lactic acid were used as de-structuring agents for SA and CS, respectively. The chemical structures, thermal stability, phase morphology, mechanical properties, water resistance, film opacity, film color, and soil burial test of thermo-compressed SA/CS films were investigated. The results indicate that intermolecular interactions in polyelectrolyte complexes in SA/CS blends were detected. Blending with CS improved the thermal stability of SA-based films. The SA/CS films showed excellent phase compatibility between SA and CS. The addition of CS improved the tensile properties of the SA-based films. The incorporation of CS in SA films resulted in enhanced water resistance and opacity and a decrease in biodegradability under soil burial. Thermo-compressed SA/CS films show promise for development and increased production capacity. These films can be tailored by varying the SA/CS ratios to display different properties. This versatility makes them suitable for a range of sustainable and diverse applications, including wound dressing, drug delivery, biosorbents, and packaging.

## 1. Introduction

Significant global concerns include greenhouse gas emissions generated by the production of fossil-based polymers and the environmental pollution caused by plastic waste [1,2]. This situation has spurred increased interest in researching and developing various biopolymers as alternatives to these fossil-based materials. Biopolymers are both biodegradable and bio-based, derived from sustainable and renewable resources [3]. Additionally, they have a lower carbon footprint compared to fossil-based polymers [4].

Alginate is an anionic polysaccharide and a biopolymer derived from brown algae, existing as the sodium salt of alginic acid [5]. Sodium alginate (SA)-based materials have garnered considerable interest in diverse applications beyond the food industry, especially in biomedical domains such as wound dressing [6], drug delivery [7,8], and tissue engineering [9]. They are also being looked into for use in water treatment [10] and packaging [11,12]. This popularity is attributed to the properties of SA, which include being edible, non-toxic, biodegradable, and low-cost, and possessing excellent film-forming capabilities [5]. SA-based materials have been developed using various techniques. These include film-forming solution [13], wet spinning [14], 3D printing [15], and thermo-compression processes [16,17]. The SA films exhibit low mechanical properties and demonstrate high solubility in aqueous environments. To address these limitations, blending various additives and polymers can be an effective solution [5].

Chitosan (CS) is a cationic polysaccharide and a biopolymer that originates from the partial deacetylation of chitin, which is the primary component of crustacean exoskeletons, such as those of crabs and shrimp [18,19]. CS possesses several beneficial properties, such as being biodegradable and non-toxic, exhibiting favorable mechanical characteristics, demonstrating excellent film-forming abilities, and showcasing antimicrobial properties [20,21,22,23]. Consequently, CS has been extensively researched and developed for various applications, including drug delivery [24], wound dressing [25], tissue engineering [26,27], water treatment [28], controlled-release fertilizers [29], and food packaging [20,21].

Blending anionic SA with cationic CS chains leads to the formation of polyelectrolyte complexes through ionic interactions [30]. The carboxylate (–COO^−^) groups on alginate are expected to interact ionically with the protonated amine (–NH_3_^+^) groups on chitosan, resulting in physical cross-linking. This interaction produces SA/CS polyelectrolyte complexes, which can be characterized as three-dimensional gels. The formation of SA/CS complexes enhances their mechanical properties and water resistance, making them more suitable for applications such as wound dressing [31,32] and food packaging [33,34]. The physical cross-linking present in SA/CS gels allows sustained drug release [35]. SA/CS films have shown effective removal of cadmium and lead from water, demonstrating greater reuse potential compared to SA and CS films. This improved performance is attributed to the formation of SA/CA complexes [10]. The preparation of SA/CS blends has been documented exclusively using the solution blending technique [31,33,34,35]. This dependence on a single method poses a significant limitation for their industrial production.

Previous research has shown that plasticized SA [16,17] and plasticized CS films [36,37] can be created using the thermo-compression technique. In this method, glycerol acts as a de-structuring agent for SA, while lactic acid is utilized for CS during the production of their thermo-compressed films. The glycerol and lactic acid in the film act as non-volatile plasticizers for SA and CS films, respectively, making them more flexible. Nevertheless, there have yet to be any reported instances of preparing SA/CS films using the thermo-compression technique, which opens up the potential for increasing the production capacity of SA/CS films at an industrial level for various applications. The study examined the influence of different SA/CS ratios on various attributes, including physicochemical properties, thermal stability, phase morphology, mechanical properties, water resistance, film opacity, film color, and biodegradation of the SA/CS films.

## 2. Results and Discussion

### 2.1. FTIR Analysis

**Figure 1:**
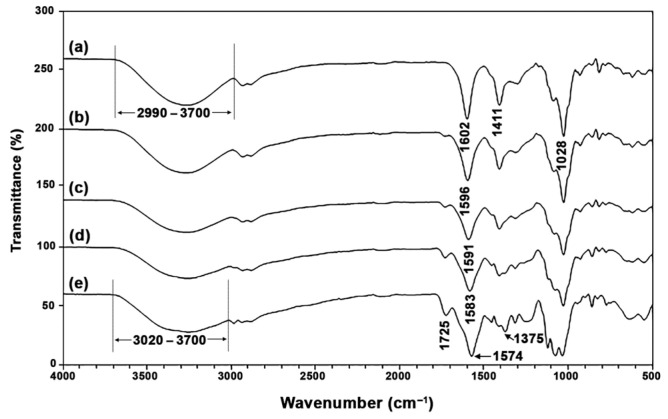
ATR-FTIR spectra of thermo-compressed SA/CS films with SA/CS ratios of (a) 100/0, (b) 90/10, (c) 80/20, (d) 60/40, and (e) 0/100 (*w*/*w*).

Figure 1 displays the ATR-FTIR spectra of film samples. This figure is utilized to identify the functional groups present in each film component, as well as to examine the intermolecular interactions among those components. The ATR-FTIR spectrum of SA films in Figure 1a shows an absorption band in the range of 2990 cm^−1^ to 3700 cm^−1^, which is attributed to the stretching of the hydroxyl (O–H) groups in SA and glycerol [16]. Additionally, the absorption bands at 1411 cm^−1^ and 1602 cm^−1^ correspond to the symmetric and asymmetric stretching of carboxylate (–COO^−^) groups, respectively [38,39]. The absorption band at 1028 cm^−1^ is associated with the asymmetric stretching of C-O-C bonds [10]. Figure 1e presents the ATR-FTIR spectrum of CS films, which displays various absorption bands corresponding to different functional groups. A broad absorption band in the range of 3020 cm^−1^ to 3700 cm^−1^ is associated with the stretching of O–H and N–H groups [37,40]. The absorption band at 1574 cm^−1^ corresponds to C–N stretching and N–H bending (amide II) in chitosan [41,42]. The absorption band at 1375 cm^−1^ is attributed to the symmetric deformation of CH_3_ groups [34]. The absorption band at 1725 cm^−1^ is associated with the stretching of C=O groups in lactic acid present in CS films [36].

The ATR-FTIR spectra of SA/CS films exhibit absorption bands that are characteristic of both SA and CS functional groups. The intensity of these absorption bands varies according to the SA/CS ratio. Intermolecular bonding between SA and CS can be identified by the shifting of the –COO band, initially observed at 1602 cm^−1^ for the SA component. For a CS ratio of 10 wt%, this –COO band appears at 1596 cm^−1^, at 1591 cm^−1^ for 20 wt%, and at 1583 cm^−1^ for 40 wt%. The observed shift toward lower wavenumbers suggests intermolecular interactions between SA and CS [10].

### 2.2. Thermal Stability

**Figure 2:**
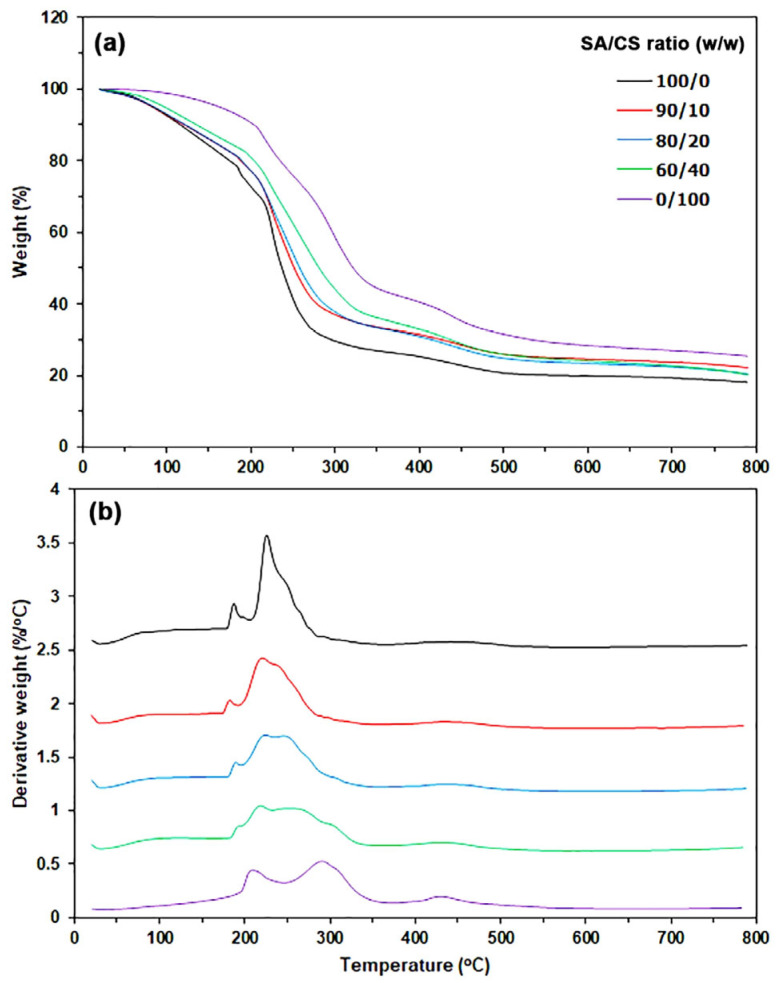
(**a**) TG and (**b**) DTG thermograms of thermo-compressed SA/CS films with varying SA/CS ratios.

Figure 2 presents the thermogravimetric (TG) and derivative TG (DTG) thermograms of the film samples. The TGA results are summarized in Table 1. The SA film exhibits two stages of thermal decomposition: one occurring in the temperature range of 50–200 °C, associated with the evaporation of moisture and glycerol, and the other in the range of 200–500 °C, related to the thermal decomposition of alginate [39]. The SA film displayed decomposition temperatures as follows: a 5% weight loss (T_5%_) at 83 °C, a 10% weight loss (T_10%_) at 116 °C, and a 50% weight loss (T_50%_) at 234 °C, as observed from the TG thermogram. It is believed that the weight losses observed at T_5%_ and T_10%_ are attributed to the evaporation of moisture. From the DTG thermogram, the SA film exhibits two temperatures at maximum weight-loss rate (T_max_) values: 187 °C and 225 °C. These T_max_ peaks are believed to represent the evaporation of glycerol and the thermal decomposition of alginate, respectively.

The TG thermogram of the CS film reveals three distinct stages of thermal decomposition, specifically within the temperature ranges of 50–150 °C, 150–250 °C, and 250–500 °C. These stages correspond to the evaporation of moisture, the evaporation of lactic acid, and the thermal decomposition of chitosan, respectively [37,43,44,45]. The CS film exhibited a T_5%_ at 162 °C, a T_10%_ at 204 °C, and a T_50%_ at 322 °C. The CS film had two T_max_ peaks, one at 216 °C and the other at 297 °C. These peaks were caused by the lactic acid evaporating and the thermal decomposition of chitosan, respectively. The high T_5%_ of the CS film at 162 °C suggests that it has a lower moisture content compared to the SA film.

The T_5%_ and T_10%_ values of SA/CS films increased with higher CS ratios, suggesting that these blend films had a lower moisture content than the SA film. This finding can be clarified by examining the T_5%_ and T_10%_ values of SA and CS films. The CS films exhibit lower moisture content compared to the SA films. Further detail will be provided in the upcoming section on the analysis of water-resistant properties. The T_50%_ values of SA/CS films were significantly higher than those of SA films, and these values increased with a higher CS ratio. This result was anticipated, as the T_50%_ value for CS film (322 °C) exceeds that of SA film (234 °C). Consequently, the T_50%_ values for SA/CS films increased with higher ratios of CS.

The data findings indicate that SA/CS films demonstrate greater thermal stability than SA films. The temperature at which glycerol evaporation peaked in the SA film was determined to be 187 °C. The blend films with 20 wt% and 40 wt% CS exhibited T_max_ peaks of 191 °C and 193 °C, respectively. The observed change is likely due to the co-evaporation of lactic acid, which has a T_max_ value of 261 °C in the CS films. The T_max_ peaks of the SA/CS films were consistent, ranging from 224 °C to 225 °C, which corresponds with the T_max_ peak observed in the SA film (225 °C). Upon closer examination of the DTG peaks for the SA/CS films presented in Figure 2b, new broad DTG peaks are observed between the DTG peak of the SA component (225 °C) and that of the CS component (297 °C). This finding corresponds with the increase in the T_50%_ value of SA/CS films as the ratio of CS increases, as previously noted. This phenomenon likely arises from the interactions between SA and CS, which lead to the formation of SA/CS polyelectrolyte complexes. These complexes exhibit enhanced thermal stability compared to the SA component [10,33].

### 2.3. Phase Morphology

**Figure 3:**
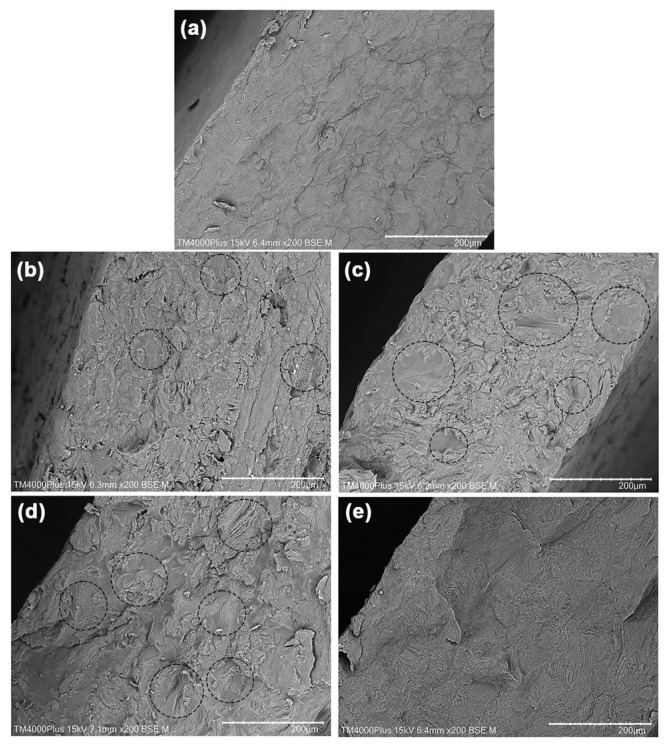
SEM images of cryofractured surfaces of thermo-compressed SA/CS films with SA/CS ratios of (**a**) 100/0, (**b**) 90/10, (**c**) 80/20, (**d**) 60/40, and (**e**) 0/100 (*w*/*w*). Some dispersed CS phases are indicated in black circles. All bar scales = 200 µm.

Phase morphology of the film samples was examined using SEM images of cryofractured film surfaces, as illustrated in Figure 3. The SA film depicted in Figure 3a presents a dense texture and a relatively smooth fracture surface, suggesting that the films are brittle. The SA particles are barely visible within the films, indicating that the glycerol-plasticization process used in this study achieved complete plasticization. Literature suggests that glycerol acts as an effective de-structuring agent in the production of plasticized SA films [16,17,39]. In contrast, the CS films illustrated in Figure 3e exhibit a rough fracture surface, which indicates that these films elongated prior to breaking. Similar to the SA films, CS particles are also not visible, suggesting that lactic acid serves as an effective de-structuring agent in the production of plasticized CS films [36,37]. The differing fracture surface textures between the SA and CS films may imply that CS films are more flexible than SA films, aligning with findings from previous research [37]. This assertion will be further validated by examining the mechanical properties of the sample films in the subsequent section.

The fracture surfaces of SA/CS films were found to be rougher than those of SA films, suggesting that SA/CS films exhibit greater flexibility. In certain areas, striated fracture surfaces are visible, highlighted by the black circles, which likely represent CS-rich phases that increase in abundance with a higher CS ratio. Nonetheless, the CS-rich phases were well dispersed within the SA film matrix, and there were no noticeable gaps between the SA matrix and CS-rich phases. SEM analysis revealed that the SA/CS films demonstrated excellent phase compatibility between the SA and CS. Furthermore, the results of this phase morphology study indicate that the mixture of glycerol and lactic acid has the potential to be used as a destructuring agent in the preparation of SA/CS blend pellets that can be further molded using thermo-compression. This finding represents a notable advancement in biopolymer blend research, especially regarding the use of different destructuring agents beyond those prepared via the solution blending method.

### 2.4. Mechanical Properties

**Figure 4:**
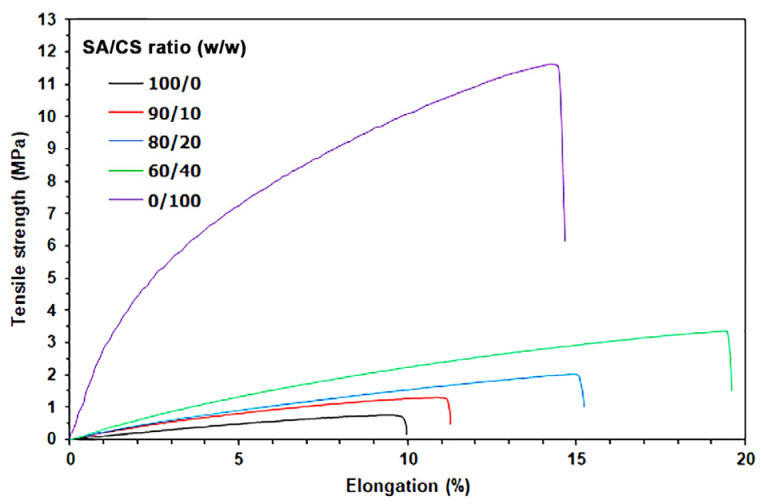
Tensile curves of thermo-compressed SA/CS films with varying SA/CS ratios.

The mechanical properties of the film samples were assessed using tensile curves, as illustrated in Figure 4. Table 2 shows a summary of the tensile properties, which include maximum tensile strength, elongation at break, and Young’s modulus. The SA films had a maximum tensile strength of 0.76 MPa, an elongation at break of 9.98%, and a Young’s modulus of 8.42 MPa. The CS films, on the other hand, had a much higher tensile strength of 11.51 MPa, an elongation at break of 14.72%, and a Young’s modulus of 110.84 MPa. The presence of amino groups in the CS films facilitates the formation of stronger hydrogen bonds, resulting in enhanced tensile properties compared to the SA films [46,47,48].

The CS blend produced SA/CS films with greater maximum tensile strength, elongation at break, and Young’s modulus compared to the SA film. The values showed a significant increase as the ratio of CS was raised. The findings suggest that adding CS enhanced the mechanical properties of thermo-compressed SA films. These phenomena are a result of the ionic interactions that occur between the negatively charged SA and the positively charged CS polyelectrolytes, which lead to the formation of a polyelectrolyte complex [5]. The elongation at break of the CS film was 14.72%, while the SA/20%CS and SA/40%CS films outperformed it at 15.21% and 19.45%, respectively. The findings that show increased elongation at breaks in the SA/CS film align with the results reported by Meng et al. [49], who prepared SA/CS blend films using a film-forming solution method. The results of the tensile tests indicate that the thermo-compressed SA/CS films produced in this study yield SA/CS polyelectrolyte complexes comparable to those formed using the film-forming solution method.

### 2.5. Moisture Absorption, Surface Wettability, and Water Dissolution

**Figure 5:**
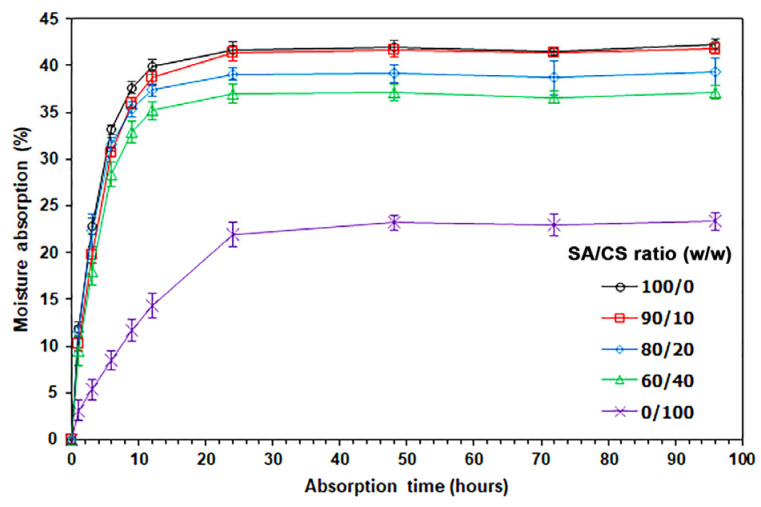
Moisture absorption of thermo-compressed SA/CS films with varying SA/CS ratios.

Figure 5 presents a graph illustrating moisture absorption values in relation to absorption time, tested at 75% RH. The sample films exhibited an increase in moisture absorption over time. All sample films maintained stable moisture absorption values after 24 h of absorption time. The moisture absorption values for the SA and CS films after 96 h were measured at 42.24% and 23.34%, respectively. The carboxylate groups (–COO^−^) in SA exhibit greater hydrophilicity compared to the OH and NH_2_ groups found in chitosan [32], which may help explain these findings. For SA/CS films, moisture absorption values decreased as the ratios of CS increased. The SA/CS films with CS ratios of 10%, 20%, and 40% demonstrated moisture absorption values of 41.78%, 39.37%, and 37.14%, respectively, after 96 h. The results suggest that the incorporation of CS into SA formulations decreases moisture absorption, likely due to the lower moisture retention properties of the CS component.

**Figure 6:**
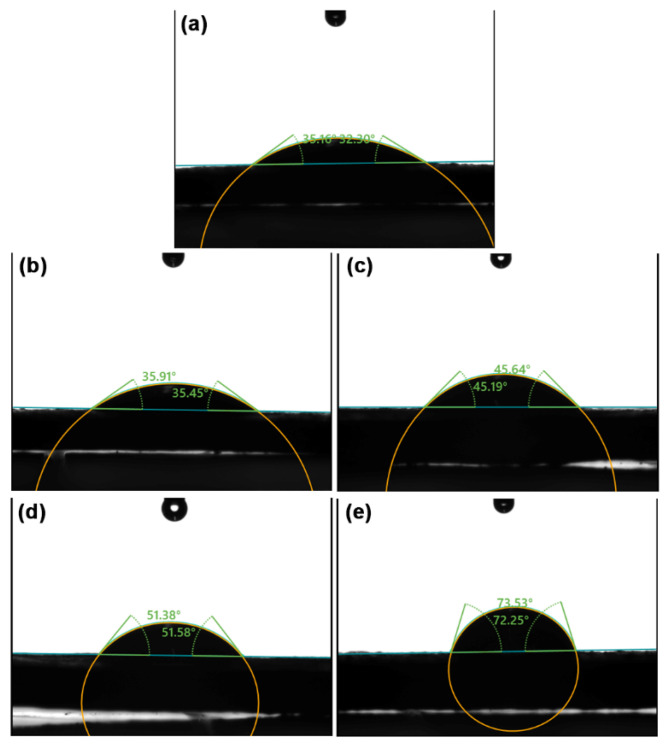
Water contact angles of thermo-compressed SA/CS films with SA/CS ratios of (**a**) 100/0, (**b**) 90/10, (**c**) 80/20, (**d**) 60/40, and (**e**) 0/100 (*w*/*w*).

Figure 6 presents the water contact angles on the film surfaces, which were analyzed to assess the surface wettability of the film samples. The average water contact angles are compiled in Table 3. A larger water contact angle indicates lower surface wettability or greater water resistance. SA and CS films exhibit water contact angles of 32.65° and 71.82°, respectively. This indicates that the SA film has higher surface wettability, or lower water resistance, compared to the CS film. This finding aligns with the research conducted by Kong et al. [50] and Wang et al. [51], who prepared these films using the film-forming solution technique. An increase in the CS ratio significantly raised the water contact angles of the thermo-compressed SA/CS films.

The water dissolution test evaluates the dissolution of film samples in water at 25 °C over a period of 24 h. This test is utilized to assess the water resistance of the film samples. The results of the water dissolution test are summarized in Table 3. SA film is fully water-soluble, exhibiting a water dissolution value of 100%. In contrast, the CS film has a significantly lower water dissolution value of only 9.14%. This characteristic can be attributed to the strong hydrogen bonds present in the CS film [32]. The water dissolution rates of SA/CS films were consistently lower than those of SA films, and the values decreased as the amount of CS increased. This decline is likely attributed to the higher content of poorly water-soluble CS. The 60/40 (*w*/*w*) SA/CS film showed a water dissolution value of only 48.61%. This result indicates that the dissolution fraction was lower than the 60 wt% SA used in its preparation. This finding suggests that the formation of a polyelectrolyte complex within the SA/CS blend may have significantly reduced the solubility of the SA phases [52].

The experimental results on moisture absorption, surface wettability, and water dissolution confirmed that the water-resistant properties of thermo-compressed SA/CS films were improved by increasing the CS content, similar to the improvement in water resistance of SA/CS films prepared by the film-forming solution technique [10,34,35]. Therefore, by varying the CS content, thermo-compressed SA/CS films can control their water resistance. The water resistance of SA/CS blends must be adjusted based on specific application requirements. For instance, these blends are suitable for controlled-release applications involving active ingredients such as drugs and fertilizers [35,53]. The reduced hydrophilicity and water solubility of SA/CS blends can be achieved by slowing the release rate of these active ingredients.

### 2.6. Film Thickness, Film Opacity, and Film Color

Table 4 provides information on the thickness, opacity, and color parameters of the film samples. The thickness of the SA film (1.11 mm) is greater than that of the CS film (0.88 mm), which suggests that the melt viscosity of the CS pellets is lower than that of the SA pellets. The thermo-compression technique used for preparing SA/CS films allows for the production of films with controllable thickness, making it a more suitable method for applications compared to the film-forming solution technique.

**Figure 7:**
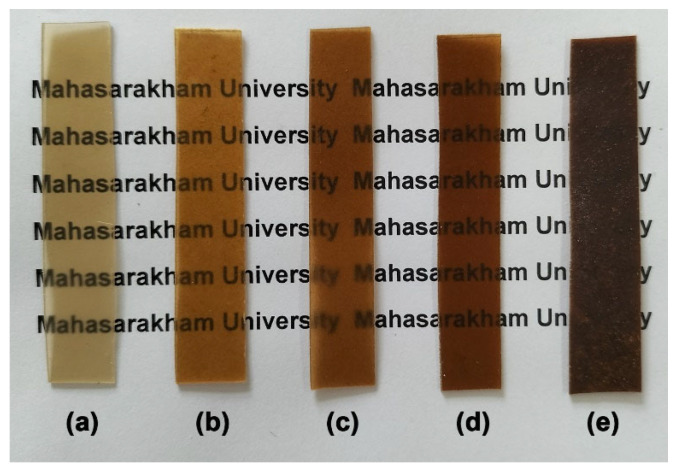
Visual transparency of thermo-compressed SA/CS films with SA/CS ratios of (**a**) 100/0, (**b**) 90/10, (**c**) 80/20, (**d**) 60/40, and (**e**) 0/100 (*w*/*w*).

The opacity of SA film (0.77 mm^−1^) is lower than that of the CS film (2.35 mm^−1^), indicating that the SA film is more transparent than the CS film. For SA/CS films, the opacity value increases with a higher CS ratio, which is attributed to the greater presence of opaque CS phases. This finding aligns with the photographs of the sample films presented in Figure 7, which show a decline in clarity as the CS ratio increases. As the CS ratio increases, it becomes more challenging to read characters beneath the film; however, characters remain legible in all the SA/CS films. The opacity of packaging materials is an important attribute for consumers, as transparent films enable visual evaluation of food freshness and appearance prior to purchase [54]. Opacity is a critical factor that must be considered alongside other characteristics, such as mechanical and barrier properties, to ensure the packaging’s suitability for its intended application.

The color of the film samples was analyzed using specific color parameters: the L* value, which measures lightness on a scale from black (0) to white (100); the a* value, which ranges from green (−120) to red (+120); and the b* value, which ranges from blue (−120) to yellow (+120). The L* values for the SA film (36.57) were higher than those for the CS film (25.16). This data indicates that the lightness of the SA film is greater than that of the CS film, which is supported by the photographs presented in Figure 7. The a* values of SA films appeared to rise with the mixing of CS, and as the CS ratios increased, suggesting that the color of the SA/CS films shifted toward the redder zone. The b* value indicated that the SA film exhibited a higher yellow color intensity (9.59) compared to the CS film (5.80). The yellow color intensity increased most significantly when mixed with 10 wt% CS, resulting in a b* value of 15.91. However, the yellow color of the SA/CS films decreased as the b* value declined when the CS ratios increased to 20 wt% and 40 wt%.

### 2.7. Biodegradation Test

**Figure 8:**
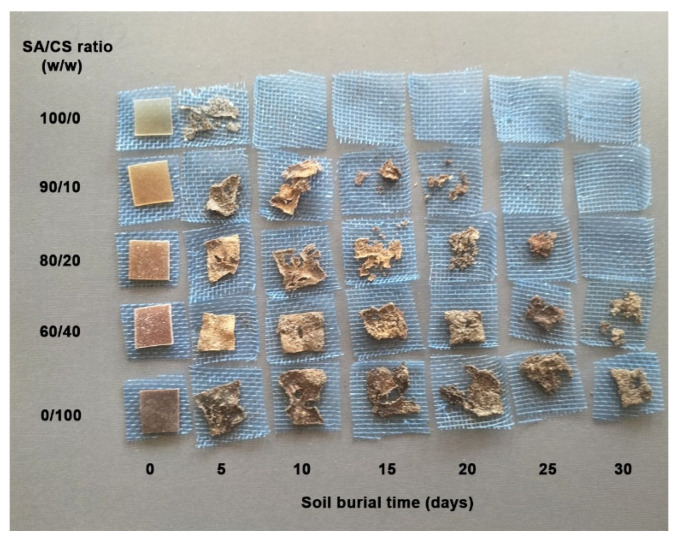
Photographs of thermo-compressed SA/CS films before and after burial in soil with varying SA/CS ratios.

Figure 8 shows photographs of film samples taken before and after burial tests. SA films completely degraded within just 10 days of burial. This conclusion is because SA is highly hydrophilic and is rapidly attacked by soil microorganisms [55]. The biodegradation of alginate under soil burial is thought to occur through alginate lyase enzymes present in various soil bacteria, including *Pseudoalteromonas*, *Pseudomonas*, and *Bacillus* [56,57]. The glycosidic bonds of alginate chains are broken down into smaller sugars (oligosaccharides and monosaccharides). These bacteria then utilize these small sugars as carbon and energy sources. CS films were found to be incompletely degraded after 30 days of burial, which may be attributed to their higher hydrophobicity compared to SA [50,51]. This conclusion is supported by the moisture absorption, surface wettability, and water dissolution analyses presented in the previous section of this research, along with CS’s anti-microbial properties. These factors therefore cause CS to degrade more slowly upon burial than SA. The biodegradation of chitosan in soil is carried out by microorganisms found in the environment [45]. It is expected that chitosan is biodegraded in soil by chitosanases, which break down the β-1,4 bonds of chitosan into smaller units, such as chitobiose and glucosamine, which microorganisms can use as carbon and nitrogen sources [58,59].

SA/CS films were found to degrade more slowly upon burial than SA films. SA/CS films degraded more slowly as the CS ratio increased, due to the increased water-resistant properties in the previous section of this research. In addition, the formation of a polyelectrolyte complex of thermo-compressed SA/CS blends with ionic interactions also contributes to the slower degradation of SA/CS films [60]. Therefore, the biodegradation rate of SA/CS films can be tailored to suit each application by varying the CS content. The results shown in Figure 8 indicate that the SA/CS films remain biodegradable, making them suitable as eco-friendly materials for various applications. The rate of biodegradation of these films plays a crucial role in their applications. Specifically, the rate at which biopolymer-based films decompose directly affects the release of fertilizers from fertilizer-loaded film matrices. A faster degradation rate leads to a quicker release of fertilizers, while a slower degradation rate provides prolonged maintenance [53].

## 3. Conclusions

Sodium alginate/chitosan (SA/CS) films were successfully produced using a thermo-compression technique. During the film formation process, glycerol served as a de-structuring agent for SA, while lactic acid played a similar role for CS. Glycerol and lactic acid are also non-volatile plasticizers for SA and CS, respectively. FTIR analysis indicated that the asymmetric stretching bands of carboxylate (–COO^−^) groups shifted to lower wavenumbers. This finding indicates the presence of intermolecular interactions within the SA/CS films. According to TGA analysis, the thermal stability of the SA films improved with the blending of CS and with an increased CS ratio. The SA/CS films exhibited a dense texture and showed good phase compatibility between the SA and CS components, as demonstrated by the SEM analysis. The incorporation of CS led to an enhancement in the maximum tensile strength, elongation at break, and Young’s modulus of the SA films. The water-resistant properties of the SA films improved, as evidenced by a reduction in moisture absorption, surface wettability, and water dissolution with increasing CS ratios. Higher ratios of CS correspondingly reduced the biodegradation rate of SA-based films when they were buried in soil. SA films were observed to have increased opacity, and the color parameters indicated a shift in the film’s color toward redder and yellower zones when CS was blended. This study concluded that SA/CS films produced using the thermo-compression technique can effectively create SA/CS blends that form polyelectrolyte complexes, similar to those made with the film-forming solution method. Thermo-compressed SA/CS films can have their properties controlled and adjusted based on the ratios of SA to CS.

Therefore, there is potential for further industrial-scale development of these films for various applications, including wound dressing, controlled release of active compounds, biosorbents, and packaging. The SA/CS blend pellets were first prepared by hand kneading and rolling. It is expected that this step can be further improved by conventional mixers, such as internal mixers and extruders. For film formation, SA/CS blend films could be successfully prepared using conventional processing techniques such as thermo-compression. However, further studies on this process are crucial in the future, particularly regarding energy consumption, film consistency, repeatability, and cost comparison with solution casting processes. Furthermore, specific applications in each field require more details about certain properties. For example, biomedical applications require studying the cytotoxicity and biocompatibility of these blend films; biosorbent applications necessitate examining the effect of system pH on adsorption; and packaging applications involve investigating barrier properties, including the oxygen, carbon dioxide, and water vapor permeability of these blend films.

## 4. Materials and Methods

### 4.1. Materials

Sodium alginate (SA), derived from seaweeds, is characterized by particle sizes smaller than 170 mesh and a viscosity of 890 cps, as measured in a 1% solution at 20 °C using a Brookfield DV3T viscometer (No. 62, 20 rpm). This product was obtained from Chanjao Longevity Co., Ltd. (Bangkok, Thailand). According to the manufacturer’s specifications, the volatile organic compound content, tested at 105 °C for 4 h, is 13.50 wt%. Additionally, the calcium content is 0.28 wt%, and the heavy metal content is less than 15 ppm. Chitosan powder prepared from shrimp shells, which has a 94% degree of deacetylation and a molecular weight of 5.4 × 10^5^ Dalton, was obtained from Sinudom Agriculture Ltd., Part. (Surathani, Thailand). The chitosan powder was sieved using a 200-mesh sieve. Glycerol, with a purity of 99.5%, was obtained from QReC Chemicals (Chonburi, Thailand). A solution of L-lactic acid with a concentration of 88 wt% was acquired from Purac (Rayong, Thailand).

### 4.2. Preparation of Thermo-Compressed SA/CS Films

**Figure 9:**
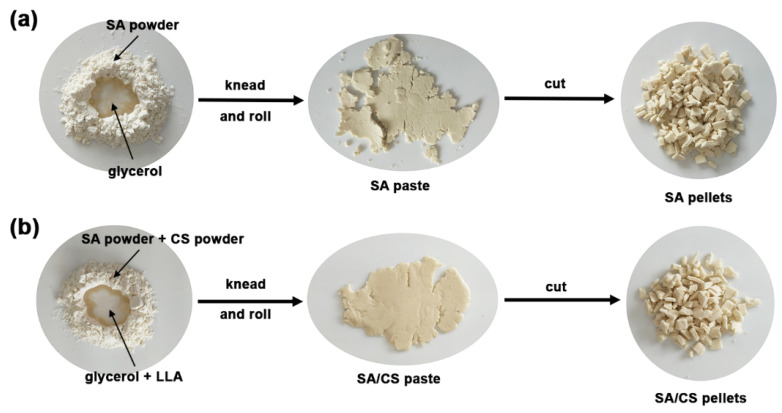
Preparation of (**a**) SA and (**b**) SA/CS pellets.

To prepare SA pellets, a mixture of SA powder and glycerol was kneaded and rolled until a uniform paste was achieved. This paste was then cut into pellets using scissors, as illustrated in Figure 9a. To prepare SA/CS pellets, a mixture of SA and CS powders, along with a glycerol/lactic acid mixture, was kneaded and rolled until a uniform paste was achieved. This paste was then cut into pellets using scissors, as illustrated in Figure 9b. The specifications for each formulation of SA and the SA/CS pellets are presented in Table 5. Glycerol content is 30 wt% based on the weight of SA, while lactic acid content is 50 wt% based on the weight of CS.

The SA and SA/CS pellets underwent thermo-compression at a temperature of 120 °C for 5 min and a compression force of 2 MPa utilizing an Auto CH Carver hot-press machine (Wabash, IN, USA). After this procedure, cooling plates were utilized to cool the films for an additional 5 min while maintaining a compressed force of 5 MPa. The films were stored for 14 days at a temperature between 25 °C and 30 °C, with a relative humidity (RH) maintained between 50% and 60%, prior to characterization [37].

### 4.3. Characterization of Thermo-Compressed SA/CS Composites

#### 4.3.1. Infrared Spectroscopy

The chemical structures of the film samples were analyzed using a Fourier transform infrared (FTIR) spectrometer (Invenio-S, Bruker, Karlsruhe, Germany) that was equipped with a diamond for attenuated total reflection (ATR). The ATR-FTIR spectra were obtained in a wavenumber range of 500 to 4000 cm^−1^. The analysis involved an accumulation of 32 scans and a resolution of 4 cm^−1^.

#### 4.3.2. Thermal Stability

A thermogravimetric analyzer (TGA, SDT Q600, TA-Instruments, New Castle, DE, USA) was employed to evaluate the thermal stability of the film samples. The analysis was conducted with a nitrogen flow rate of 100 mL·min^−1^, and the heating rate was set at 20 °C·min^−1^.

#### 4.3.3. Phase Morphology

A scanning electron microscope (SEM, TM4000Plus, Hitachi, Tokyo, Japan) was utilized, operating at a voltage of 15 kV, to examine the phase morphology of the film cross-sections. The film samples were cryo-fractured in liquid nitrogen and then sputter-coated with gold before analysis.

#### 4.3.4. Mechanical Properties

The tensile properties of the film samples were evaluated using a universal testing machine (LY-1066B, Dongguan Liyi Environmental Technology Co., Ltd., Dongguan, China). This machine was equipped with a 100 kg load cell and operated at 25 °C. The samples for tensile testing were cut into strips that were 60 mm by 10 mm. The initial gauge length was established at 40 mm, and the crosshead speed was set to 50 mm·min^−1^. For each sample, five films were evaluated, and both the average values and the standard deviation (SD) were recorded.

#### 4.3.5. Moisture Absorption

The film samples (20 mm × 20 mm) were dried in an air oven at 105 °C for 24 h before being weighed (W_1_). The film samples were maintained in a desiccator at 75% RH. At specified intervals, the film samples were measured for weight (W_2_). The calculation of moisture absorption for the film samples was performed using the equation provided below. Each sample was tested on three specimens, and the average ± SD values are given.

Moisture absorption = [(W_2_ − W_1_)/W_1_] × 100% (1)

#### 4.3.6. Water Dissolution

The film samples (20 mm × 20 mm) were subjected to drying in an air oven at 105 °C for 24 h before being weighed (W_3_). The film samples were then immersed in 50 mL distilled water and agitated at 100 rpm for 24 h at 25 °C. The film samples were subsequently dried in an air oven at 105 °C for 24 h before being weighed (W_4_). The dissolution of the film samples in water was assessed using the equation provided below. Three specimens for each sample were tested, and the mean and SD values are reported.

Water dissolution = [(W_3_ − W_4_)/W_3_] × 100% (2)

#### 4.3.7. Surface Wettability

The sessile drop technique was used to determine the surface wettability of the film samples using a contact angle analyzer (OCA11, DataPhysics Instruments, Germany). At 15 s after the 2.5 µL water was dropped, the water contact angle on the film surface was measured from the left and right contact angles, and it was then averaged. Three measurements were taken for each sample, and the mean and SD values are reported.

#### 4.3.8. Film Thickness and Film Opacity

A digital micrometer (Mitutoyo, Kanagawa, Japan) was utilized to measure the thickness of the film samples with a precision of 0.001 mm. A UV-Vis spectrophotometer (Cary 60, Agilent Technologies, Mulgrave, VIC, Australia) measured the absorbance at a wavelength of 600 nm (A_600_) to assess the opacity of the film samples. The following equation was used to determine the film opacity [61]. The assay was performed on three films for each sample, and the mean and SD values are reported.

Film opacity (mm^−1^) = A_600_/X (3)

where X is the thickness of the film sample (mm).

#### 4.3.9. Film Color

The color of the film samples was evaluated using a color spectrophotometer (UltraScan Vis, HunterLab, VA, USA). This device was equipped with a D65/10° illuminant observer. The CIELAB color space consists of three components: lightness (L*), red-green (a*), and yellow-blue (b*). Three samples of each film were analyzed, and the average results were calculated with the SD.

#### 4.3.10. Soil Burial Test

The soil burial test was used to assess the biodegradability of film samples. The film samples (15 mm × 15 mm) were prepared and placed into nylon mesh bags with 1.0 mm mesh before burying 5 cm below the soil surface. The soil was watered every other day. The pH value was kept between 6.0 and 7.0, while the moisture value was maintained between 50% and 70%. At predetermined intervals, the film samples within the nylon mesh bags were visually inspected to determine the level of biodegradation.

### 4.4. Statistical Analysis

One-way ANOVA was employed to analyze the experimental data, followed by Duncan’s post hoc test. The results indicate statistically significant differences at *p* < 0.05 with SPSS version 22.0, presented as mean ± SD.

## Figures and Tables

**Table 1:** Thermal stability of thermo-compressed SA/CS films.

SA/CS Ratio (*w*/*w*)	T_5%_ (°C) ^1^	T_10%_ (°C) ^1^	T_50%_ (°C) ^1^	T_max_ (°C) ^2^
100/0	83	116	234	187, 225
90/10	83	121	252	187, 225
80/20	85	123	258	191, 225
60/40	97	136	281	193, 224
0/100	102	204	322	216, 297

^1^ Obtained from TG thermograms. ^2^ Obtained from DTG thermograms.

**Table 2:** Tensile properties of thermo-compressed SA/CS films.

SA/CS Ratio (*w*/*w*)	Maximum Tensile Strength (MPa)	Elongation at Break (%)	Young’s Modulus (MPa)
100/0	0.76 ± 0.24 ^a^	9.98 ± 1.36 ^a^	8.42 ± 2.34 ^a^
90/10	1.31 ± 0.16 ^b^	11.28 ± 3.41 ^a^	10.75 ± 1.82 ^a^
80/20	2.04 ± 0.32 ^c^	15.21 ± 2.86 ^b^	17.56 ± 2.65 ^b^
60/40	3.35 ± 0.47 ^d^	19.45 ± 3.52 ^c^	25.84 ± 3.82 ^c^
0/100	11.51 ± 1.45 ^e^	14.72 ± 2.16 ^b^	110.84 ± 8.27 ^d^

Values are shown as mean ± standard deviation (*n* = 3). Column values represented by the letters (a, b, c, d, and e) demonstrate substantial differences (*p* < 0.05).

**Table 3:** Water contact angle and water dissolution of thermo-compressed SA/CS films.

SA/CS Ratio (*w*/*w*)	Water Contact Angle (°)	Water Dissolution (%)
100/0	32.65 ± 2.14 ^a^	100.00 ± 0.00 ^e^
90/10	35.21 ± 2.58 ^a^	96.00 ± 3.62 ^d^
80/20	44.68 ± 3.54 ^b^	83.09 ± 2.20 ^c^
60/40	52.64 ± 2.86 ^c^	48.61 ± 3.21 ^b^
0/100	71.82 ± 4.51 ^d^	9.14 ± 1.43 ^a^

Values are shown as mean ± standard deviation (*n* = 3). Column values represented by the letters (a, b, c, d, and e) demonstrate substantial differences (*p* < 0.05).

**Table 4:** Thickness, opacity, and color parameters of thermo-compressed SA/CS films.

SA/CS Ratio (*w*/*w*)	Film Thickness (mm)	Film Opacity (mm^−1^)	Color Parameters		
			L*	a*	b*
100/0	1.11 ± 0.06 ^c^	0.77 ± 0.03 ^a^	36.57 ± 0.19 ^d^	0.96 ± 0.04 ^a^	9.59 ± 0.12 ^b^
90/10	1.10 ± 0.04 ^c^	1.09 ± 0.03 ^b^	35.50 ± 0.04 ^d^	5.16 ± 0.31 ^b^	15.91 ± 0.36 ^d^
80/20	0.96 ± 0.03 ^b^	1.35 ± 0.05 ^c^	31.85 ± 0.09 ^c^	5.58 ± 0.15 ^b^	12.92 ± 0.08 ^c^
60/40	0.85 ± 0.05 ^a^	1.81 ± 0.14 ^d^	29.74 ± 0.15 ^b^	6.06 ± 0.09 ^c^	10.17 ± 0.18 ^b^
0/100	0.88 ± 0.06 ^a^	2.35 ± 0.07 ^e^	25.16 ± 0.12 ^a^	6.21 ± 0.12 ^d^	5.80 ± 0.14 ^a^

Values are shown as mean ± standard deviation (*n* = 3). Column values represented by the letters (a, b, c, d, and e) demonstrate substantial differences (*p* < 0.05).

**Table 5:** Formulation of thermo-compressed SA/CS films.

SA/CS Ratio (*w*/*w*)	SA (g)	CS (g)	Glycerol (g)	LLA (g)
100/0	10.00	-	4.29	-
90/10	9.00	1.00	3.86	1.00
80/20	8.00	2.00	3.43	2.00
60/40	6.00	4.00	2.58	4.00
0/100	-	10.00	-	10.00

## Data Availability

Data is contained within the article.
